# Acute Parenchymatous Nephritis

**Published:** 1883-04

**Authors:** John Lindsay

**Affiliations:** Huntington, L. I.


### IV.—ACUTE PARENCHYMATOUS NEPHRITIS.

BY JOHN LINDSAY, D.V.S.

On November 20th, 1882, I was first called to attend a colt in this town. He was only eleven months old, and unusually well developed for his age. After the autopsy I obtained the following previous history, which was as follows:

The colt had been subject to continual sweating, and the surrounding temperature seemed to have no influence upon it. Even in the coldest weather the perspiration flowed as if the animal had been furiously driven on a warm day. He had never been perfectly dry since the first attack of sickness. Since then he had always showed signs of slight pain, and while the pain lasted constantly bit at his loins. Had only once been seen to urinate and then passed about one-half pint. He also had a very bad smell. His appetite, however, was good, and he appeared to feel pretty well. At this time he seemed somewhat reduced in flesh for one that had been out to pasture. He had been brought to the stable on this day.

On examination I found the animal to be suffering from colicky pains, repeatedly lying down and getting up, rubbing against the stall, biting at the left hind leg, and anxiously looking at the left flank, pulse quick and strong, 80, temperature 103, with a desire to micturate but once while I was there. Food of day left in the manger.

I diagnosticated some renal trouble, and gave oleum lini. oj., Tr. opii ℥i., spiritus etheus nitrosi ℥i., also a rectal injection of warm water. Had some thick blankets wrung out in hot water and placed over the loins. This treatment gave him relief from the acute symptoms.

I remained for an hour and there was no return of pain, and he commenced to eat. When I left him I ordered a renewal of the hot blankets; also left word that in case he showed signs of returning pain to give chloral hydrate ℥ss. in Oss. warm water and to keep using the warm applications to the back. Nov. 21st colt looked bright, and was eating well ; had no return of pain.

Heard nothing further from the colt until February 2d, when the owner called to say that the horse had lost his appetite and was again suffering from pain. When I arrived at the stable the animal stood in one corner with head dependent, eyes dull and heavy. I watched him for half an hour, but he showed no signs of pain. His food was in his box. Temperature, 101°, F. Pulse, 38 to the minute.

I informed the owner that I considered his horse to be suffering from indigestion, ordered oleum lini Oss., all feed removed, and the next morning a few steamed oats.

On the 5th I was informed that the owner was much worried as the colt had a bad diarrhœa since the night ot the 3d, and was growing weak very fast.

When I examined the animal he was lying down, his ears and nose very cold. I made him get up, when he immediately began to strain, passing nothing but mucous. He also showed some signs of pain. I thought the colt to be suffering from mucous enteritis and at once gave Tr. opii ℥i., spts. etheus nitrosi ℥iss., spts. vini gallici ℥iv., in one pint of thin flour gruel, this dose to be repeated in one hour if the pain had not ceased. At the time I gave him an enema of flour gruel containing ℥i. of Tr. opii. This gave some relief. I also left orders for all water to be kept from him, and its place taken by milk and eggs.

At eight o'clock, P. M., I was informed that everything was progressing favorably, and that he had drunk twice of the milk and eggs.

At eleven o'clock, P. M., I was called, and with the summons came the information that the colt was dying.

When I arrived the colt lay upon his side with legs outstretched. No pain. Breathing short and quick. Eyes insensible to light. Mouth very cold.

Gave spts. etheus nitorsi ℥ii., Tr. digitalis ♏ xx. In fifteen minutes he got up, but a short time after dropped dead.

*Necropsy.*—The animal was well developed, being unusually large for the age, and when the skin was opened a fair amount of adipose tissue was found. The skin itself was loose, oily, clean, the hair smooth and glossy.

*Thoracic cavity.*—The heart was fine and large, but not out of proportion to the size of the animal. The internal structures being well developed, the valves free and sufficient. The arteries were large and strong. Normal amount of blood and clots in the heart cavities. The blood, however, was a little dark in color.

*Lungs.* Both of these organs were normal save a slight congestion and considerable œdema.

*Spleen.*—This organ was normal and well developed.

*Kidneys.*—Both organs were completely disorganized. They were nearly free from all perinephritic adipose tissue. They had not their normal shape, but that of a flattened mass. Their true capsules were easily torn, and when ruptured the contents, or that which once was the renal tissue, easily spread itself out into a thin, flat, gelatinous mass. The glands were not 1.5 centimetres ($\frac{1}{2}$ in.) thick at their centres. I tried to cut them, but it was impossible. The knife would go through, but there was no cut surface to be seen after it was withdrawn.

*Bladder.*—This organ had about two ounces of urine in it.

*Stomach.*—It contained about four quarts of liquid food, muscular coat slightly congested, mucous coat normal.

*Small Intestines.*—Were slightly congested both in the mucous and muscular coats. They contained some liquid food.

*Large Intestines.*—These were slightly congested, but practically normal.

*Liver.*—This organ showed very great congestion of the right lobe, with numerous large ecchymotic spots. When cut these extravisations were black, as if gangrene was about to set in. The right lobe was less markedly involved.

HUNTINGTON, L. I.

[Again we have the unfortunate fact that there is no history of any examination of the urine. We are sorry to have again, and so constantly to call attention to this important fact that examinations of the urine are so often neglected. From the fact that we have no account of the urine and kidneys too rotten for microscopical examination, it must of necessity leave the diagnosis of the case somewhat in the dark. But, judging from the rational signs, we are inclined to think that the case is one of acute parenchymatous nephritis. The case is certainly one of great interest, and shows careful observation on the part of the attending physician, and we sincerely wish there were a larger number of veterinarians who had as deep an interest in scientific medicine, and would forward their cases for publication and the good of their fellow practitioners.—ED.]

